# Panel regression analysis of the relationship between health resources and infant mortality rate in Chinese mainland provinces, 2010–2020

**DOI:** 10.3389/fpubh.2026.1916606

**Published:** 2026-09-25

**Authors:** Ruonan Gao, Hailong Li, Kun Zou, Zhenyan Bo, Yong Tang, Shaoyang Zhao, Yongmu Jiang, Lingli Zhang

**Affiliations:** 1 Department of Pharmacy/Evidence-Based Pharmacy Center, West China Second University Hospital, Sichuan University, Chengdu, China; 2 Children's Medicine Key Laboratory of Sichuan Province, Chengdu, China; 3 NMPA Key Laboratory for Technical Research on Drug Products In Vitro and In Vivo Correlation, Chengdu, China; 4 Key Laboratory of Birth Defects and Related Diseases of Women and Children, Sichuan University, Ministry of Education, Chengdu, China; 5 West China School of Medicine, Sichuan University, Chengdu, China; 6 School of Economics, Sichuan University, Chengdu, China; 7 Chinese Evidence-Based Medicine Center, West China Hospital, Sichuan University, Chengdu, China

**Keywords:** child health, China, health expenditure, health resources, infant mortality

## Abstract

**Background:**

China has substantially reduced its infant mortality rate (IMR) and targets further declines under the Healthy China 2030 blueprint. Health resources are fundamental to infant health. However, national evidence on the association between multidimensional health inputs and IMR remains limited, particularly for pediatric specific resources.

**Methods:**

A panel dataset of 31 Chinese mainland provinces (2010–2020) was analyzed. IMR was defined as infant deaths per 1,000 live births. Associations between categorized health resources and IMR were examined using fixed- and random-effects panel regression adjusted for socioeconomic covariates.

**Results:**

Mean provincial IMR was 6.70 per 1,000 live births. Most health resource indicators correlated negatively with IMR. In adjusted panel models, health technical personnel (*β* = −0.88, 95%CI −1.11, −0.66) and beds (*β* = −1.26, 95%CI −1.50, −1.02) per 1,000 population were inversely associated with IMR. Health expenditure per capita (*β* = −1.04, 95%CI −1.40, −0.69) and pediatric beds per 10,000 children (*β* = −0.39, 95%CI −0.46, −0.31) also showed significant inverse associations. Government health expenditure was not significant (*p* = 0.15).

**Conclusion:**

This study reveals a significant association between increased health workforce and facility capacity and lower infant mortality rates, highlighting the critical role of adequate pediatric-specific resource allocation. Our findings suggest that China should continue to ensure an adequate supply of health resources and potentially direct government health expenditure patterns specifically toward strengthening primary pediatric care and maternal and child health institutions.

## Background

Globally, the infant mortality rate (IMR) has decreased markedly over the past three decades, while regional disparities still exist. Data from the United Nations Inter-agency Group for Child Mortality Estimation indicates that the global IMR reached roughly 29 per 1,000 live births in 2020, with notable regional differences. The IMR was 4 per 1,000 live births in high-income countries and 32 per 1,000 live births in low- and middle-income countries (1). Asian regions have witnessed considerable improvements in IMR, albeit with persistent intraregional inequalities. China attained an extremely low IMR of 5.4 per 1,000 live births in 2020, ranking it among high-performing middle- and high-income countries worldwide (2).

Despite this national success, substantial disparities in IMR continue to exist between urban and rural areas in China. For instance, in 2023, the infant mortality rate in urban areas of China was 2.9 per 1,000 infants, while in rural areas it was 5.2 per 1,000 infants (3). The outline of the “Healthy China 2030” blueprint proposes to reduce China’s IMR to less than 5.0‰ by 2030 and narrow the geographical disparities in IMR (4). Understanding the factors that drive these residual disparities is therefore a pressing policy priority, particularly in an era of low fertility and rapid population aging, where protecting the health of every newborn has profound demographic and social implications.

Health resources, including health workforce, medical facilities and health financing, serve as the foundation of health system operation and can be directly regulated through policy measures (5–8). In China, pediatric care is primarily delivered through two parallel systems: specialized children’s hospitals and maternal and child healthcare (MCH) centers, which together form the backbone of institutional pediatric service provision. However, the distribution of these pediatric-specific resources remains highly uneven across provinces, with marked urban–rural and coastal-inland gradients (9). Previous studies have examined the ecological correlates of IMR in China, identifying factors such as health literacy, female education, and economic development. Several investigations have also linked physician density or primary care supply to infant health outcomes. Nevertheless, most existing national-level analyses have relied on cross-sectional data or examined health resources in isolation, without simultaneously considering both general health system capacity and pediatric-specific resource allocation within a unified longitudinal framework (10–12). Furthermore, although the correlation between socioeconomic factors and IMR has been well documented, the independent effects of various health resource categories remain insufficiently explored in the Chinese setting. This is particularly true for the relative importance of general health inputs and pediatric-specific resources.

This study therefore aims to address these gaps by: (1) examining the relationships between multiple dimensions of health resources and provincial IMR in China from 2010 to 2020 using panel regression techniques, and (2) comparing the relative associations of general health system resources versus pediatric-specific resources with IMR. By identifying which types of health investments are most strongly linked to IMR reduction, the findings are intended to inform evidence-based resource allocation strategies aligned with the Healthy China 2030 targets, ultimately contributing to more equitable and efficient improvements in infant survival across all Chinese provinces.

## Methods

### Study design

This study is a longitudinal ecological analysis based on unbalanced panel data from 2010–2020 for 31 provinces in mainland China. Provinces were used as the unit of analysis to minimize the association of population movements and deaths on the results. By effectively controlling for unobservable factors affecting health outcomes, panel data can be constructed with larger sample sizes over longer time horizons and have advantages in improving estimation accuracy and accounting for individual heterogeneity (5).

### Data source

Data of IMRs and health resources were obtained from the China Health Statistics Yearbook (China Health and Family Planning Statistics Yearbook) 2011–2021 and that of 31 provinces. The socio-economic data was obtained from the China Statistical Yearbook and provincial statistical yearbooks 2011–2021. To ensure temporal alignment, all socio-economic covariates were extracted from the yearbook edition corresponding to the specific year of IMR and health resource data.

### Outcome

The IMR was used as the dependent variable. IMR was defined as the ratio of the number of deaths of infants less than 1 year old to the number of live births during the year. In line with standard practice in national-level ecological studies, crude IMR was used as the outcome. While age-standardized IMR could theoretically enhance comparability, province-level data stratified by maternal age or birth order required for direct standardization are not publicly available in the China Health Statistics Yearbook. To mitigate potential bias from differences in provincial demographic structures, the fixed-effects panel model inherently controls for all time-invariant province characteristics, and time-varying socioeconomic covariates (e.g., per capita GDP, education level, birth rate) were included in the adjusted models.

### Exposures (indicators of health resources)

The target independent variables of health resources were categorized as health workforce (number of health technical personnel per 1,000 population), health facility (hospital beds per 1,000 population), and health finance investment (health expenditure per capita, government health expenditure, and total health expenditure as a percentage of gross domestic product [GDP])^1^.

There are two stream of pediatric-specific health resources in China, namely children’s hospitals and maternal and child healthcare center. Therefore, pediatric-specific health resources were also classified as health workforce (number of health technical personnel of children’s hospital per 10,000 children, number of health technical personnel of maternal and child healthcare center per 10,000 children) and health facility (number of pediatric beds per 10,000 children). Since only 2 out of 31 provinces reported data on the number of pediatric health technical personnel, health technical personnel in children’s hospitals and maternal and child healthcare centers were used as surrogate to characterize the health workforce of pediatric in each province. This approach is justified by the role of these institutions as the primary providers of pediatric care in China, making their workforce data a representative indicator of provincial pediatric health resources.

The population <14 years of age was used as the children population, as China’s census data stratifies age groups into 0–14, 15–59, and ≥60 years. This age grouping (<14 years) was dictated by the reporting structure of China’s statistical yearbooks, which do not provide province-level population data disaggregated by finer pediatric age bands. Consequently, the pediatric resource indicators represent average resource availability across the entire child population rather than infant-specific density. This limitation should be considered when interpreting the associations.

These indicators represent aggregate provincial health system capacity rather than infant-specific service delivery. Higher densities of health personnel and beds reflect greater system readiness and accessibility, creating an enabling environment that benefits all populations, including infants. The observed associations thus capture systemic rather than individual-level effects.

### Covariates

Socio-economic factors, including gross regional product per capita, birth rate, years of education per capita and urbanization rate, were selected as control variables according to literature (6, 7). Definitions and calculations of all variables were presented in Supplementary Table S1.

### Statistical analysis

Descriptive statistical analyses were performed using means and standard deviations for quantitative variables, and frequency and percentage for categorical variables. Scatter plots were made separately for each target independent variable using IMR as the horizontal coordinate. Spearman correlation analysis was used to analyze the correlation between IMR and health resources indicators. Spearman’s rank correlation was chosen as a non-parametric method that does not assume normality, providing a robust preliminary assessment given that IMR and several health resource indicators exhibited skewed distributions. Variance inflation factor (VIF) was calculated for all independent variables and covariates to assess multicollinearity. As each panel regression model included only one exposure variable at a time, the collinearity in final models was substantially lower than in a global VIF assessment.

To ensure the stability of time series data and avoid spurious regression problems, the Augmented Dickey-Fuller (ADF) test was used to test the unit roots of all variables. ADF test is a widely used statistical test method to determine whether there is a unit root in a time series data, that is, whether the sequence is stable. If the *p*-value of the ADF test is less than 0.05, the sequence is considered to be stable (8). The test is carried out by three types: no drift no trend, with drift no trend and with drift and trend.

Panel model assumptions were validated using several diagnostic tests. Heteroskedasticity was assessed using the Breusch-Pagan test; serial correlation was evaluated using the Wooldridge test for panel data; strict exogeneity was tested by adding lead terms of all explanatory variables and performing a joint *F*-test; and functional form was assessed using the Ramsey RESET test. Where violations were detected, province-clustered robust standard errors were applied to ensure valid inference.

We used panel data linear regression models to explore the associations between health resources and IMR. Panel data regression was chosen for its ability to control for unobserved province-level heterogeneity, increase degrees of freedom, and reduce collinearity among variables compared to conventional cross-sectional or time-series analyses (9). For regression models, observations with missing values for any included variable were excluded using listwise deletion. Selection of fixed effects model or random effects model was based on the results of the Hausman test (10). If the *p*-value of the Hausman test was less than 0.05, the fixed effect model was selected, otherwise the random effect model was selected. Since all explanatory variables and covariates in this study were time-varying, the standard fixed-effects model fully controls for unobserved time-invariant province heterogeneity without the additional instrumental variable assumptions required by more complex estimators such as Hausman-Taylor.

Two panel data models were built for each target independent variable. IMR was used as the dependent variable. Model 1 included only overall health resources or pediatric health resources (unadjusted), model 2 incorporates socio-economic covariates on the basis of model 1(adjusted).

The fixed effects model equation can be expressed as:

$$\mathrm{IM}R_{\mathrm{it}}=\beta_{0}+\Sigma_{j=1}^{n}\beta_{j}X_{\mathrm{jit}}+\alpha_{i}+\varepsilon_{\mathrm{it}}$$

IMR_it_ represents the IMR of the *i*^th^ province at time *t*; *X*_jit_ represents the *j*^th^ explanatory variable of the *i*^th^ province at time *t*; *β*_j_ is the coefficient corresponding to the fixed effect model; *α*_i_ is the fixed effect of the *i*^th^ province, capturing the unobservable heterogeneity across provinces; *ε*_it_ is the error term.

The random effects model equation can be expressed as:

$$\mathrm{IM}R_{\mathrm{it}}=\gamma_{0}+\Sigma_{j=1}^{n}\gamma_{j}X_{\mathrm{jit}}+u_{i}+\varepsilon_{\mathrm{it}}$$

*γ*_j_ is the coefficient corresponding to the random effects model; *u*_i_ is the random effect of the *i*^th^ province, which is assumed to be uncorrelated with the explanatory variables; the rest of the symbols have the same meaning as in the fixed effects model.

To mitigate potential reverse causality, we conducted a sensitivity analysis using one-year lagged values of all independent variables and covariates (i.e., resources and socioeconomic factors in year *t*-1 were used to predict IMR in year *t*).

All statistical tests were performed with *α* = 0.05 as the level of significance and statistical analyses were performed using R4.4.0 and SPSS Statistics26.

## Results

### Descriptive statistics

The descriptive statistics of dependent variables and independent variables from 2010 to 2020 were shown in Table 1. Taking the IMR as the *Y*-axis, health resources were used as the *X*-axis to make scatter plots, the results were shown in Supplementary Figure S1.

**Table 1:** Descriptive statistics of variables of 31 provincial-level administrative regions in China from 2010 to 2020.

Variable name	Number of province-year observations	Mean	SD
Dependent variable			
Infant mortality rate (‰)	341	6.70	4.19
Target independent variables: health resources			
Number of health technical personnel per 1,000 population	341	6.11	1.76
Number of health institution beds per 1,000 population	341	5.13	1.18
Target independent variables: health finance investment			
Health expenditure per capita (1,000 Yuan)	341	3.40	1.85
Government health expenditure (Ten billion Yuan)	333	4.07	2.77
Total health expenditure as a percentage of GDP (%)	333	6.51	1.90
Target independent variables: Pediatric-specific health resource			
Number of pediatric beds per 10,000 children	341	13.09	4.17
Number of children’s hospital health technical personnel per 10,000 children	132	4.57	5.89
Number of maternal and child healthcare center health technical personnel per 10,000 children	341	12.77	5.70
Control variables			
Gross regional product per capita (10,000 Yuan)	341	5.10	2.64
Birth rate (‰)	341	9.13	1.08
Years of education per capita (year)	341	11.09	2.80
Urbanization rate (%)	341	56.80	13.36

SD, standard deviation.

### Correlation analysis and ADF test

The results of Spearman correlation analysis between independent variables and IMR were shown in Table 2. There was a significant positive correlation between birth rate and IMR, and there was no significant correlation between the total health expenditure as a percentage of GDP and IMR. There was a significant negative correlation between the remaining variables and IMR. Global VIF analysis showed that urbanization rate (VIF = 10.81), gross regional product per capita (VIF = 18.02), and years of education per capita (VIF = 6.10) exceeded the acceptable threshold (VIF > 5). Urbanization rate was therefore excluded. Per capita GDP and education were retained as essential confounders. Among the variables included in the final models, all VIF values were below 5 (range: 2.07–4.84), confirming the absence of serious multicollinearity. The results showed that the urbanization rate was strongly correlated with gross regional product per capita and years of education per capita, so it was not suitable to be included in the model for analysis to avoid serious collinearity problems. The correlation analysis and VIF results between all variables were shown in Supplementary Tables S2, S3.

**Table 2:** Spearman correlation analysis of infant mortality rate.

Variables	Correlation coefficient	*P*
Number of health technical personnel per 1,000 population	−0.63	<0.01
Number of health institution beds per 1,000 population	−0.42	<0.01
Health expenditure per capita (1,000 Yuan)	−0.65	<0.01
Government health expenditure (Ten billion Yuan)	−0.62	<0.01
Total health expenditure as a percentage of GDP (%)	0.06	0.26
Number of pediatric beds per 10,000 children	−0.48	<0.01
Number of children’s hospital health technical personnel per 10,000 children	−0.54	<0.01
Number of maternal and child healthcare center health technical personnel per 10,000 children	−0.60	<0.01
Gross regional product per capita (10,000 Yuan)	−0.77	<0.01
Birth rate (‰)	0.39	<0.01
Years of education per capita (year)	−0.60	<0.01
Urbanization rate (%)	−0.69	<0.01

The ADF test was performed on the infant mortality rate and the target independent variables. The results showed that the *p*-values of all lag orders were less than 0.01 under the with drift no trend and with drift and trend types, which means that at the 1% significance level, the null hypothesis of the existence of unit root can be rejected. Therefore, it is suitable to use the fixed effect model and random effect model of the panel data model for analysis to include drift items and trend items. In the no drift no trend type, the partial lag order of the number of health technical personnel per 1,000 population, the number of health institution beds per 1,000 population and the total health expenditure as a percentage of GDP cannot reject the null hypothesis. The complete ADF test results are shown in Supplementary Table S4.

### Panel data model

The results of panel data model were shown in Table 3. In model 1, the increase of all health resource variables was significantly associated with the reduction of IMR (*p* < 0.05). After adding socio-economic factors, the association of health resources on IMR was weakened, and government health expenditure became non-statistically significant (*p* = 0.15). Results of panel diagnostic tests are reported in Supplementary Tables S5, S6; province-clustered robust standard errors were used throughout.

**Table 3:** Regression results of China’s health resources on infant mortality rate from 2010 to 2020.

Variables	Model 1 (unadjusted; model)			Model 2 (adjusted for socio-economic covariates)		
	Fixed effect (*t* value)	Random effect (*z* value)	Hausman test	Fixed effect (*t* value)	Random effect (*z* value)	Hausman test
Number of health technical personnel per 1,000 population	−1.63*(−17.54)	−1.59*(−17.41)	0.02	−0.88*(−7.65)	−0.89*(−7.66)	<0.01
Number of health institution beds per 1,000 population	−1.96*(−20.61)	−1.93*(−20.2)	<0.01	−1.27*(−9.9)	−1.26*(−10.44)	0.42
Health expenditure per capita (1,000 Yuan)	−1.34*(−18.25)	−1.32*(−18.2)	0.09	−1.07*(−5.34)	−1.04*(−5.76)	0.09
Government health expenditure (Ten billion Yuan)	−0.83*(−12.76)	−0.82*(−13.00)	0.43	**−0.14** ^**†**^ **(−1.44)**	−0.31*(−3.68)	<0.01
Total health expenditure as a percentage of GDP (%)	−1.57*(−17.44)	−1.29*(−13.27)	<0.01	−0.86*(−7.56)	−0.75*(−7.77)	0.05
Number of pediatric beds per 10,000 children	−0.65* (−16.57)	−0.63* (−16.35)	<0.01	−0.39* (−9.60)	−0.38* (−9.63)	<0.01
Number of children’s hospital health technical personnel per 10,000 children	−0.53*(−4.14)	−0.46* (−4.30)	0.31	−0.31* (−2.86)	−0.20 (−1.89)	0.04
Number of maternal and childcare service center health technical personnel per 10,000 children	−0.46* (−11.90)	−0.45* (−12.37)	0.47	−0.21* (−6.02)	−0.23* (−6.69)	<0.01

*p < 0.05.

^†^*p* = 0.15.

^‡^The bold body represents the model selected after Hausman test.

#### Overall health resources

In model 1, an increase of each additional health technical personnel and health institution beds per 1,000 population was associated with a decrease in IMR of 1.62‰ (95%CI −1.81, −1.45) and 1.96‰ (95%CI −2.15, −1.77), respectively. In model 2, IMR decreased by 0.88‰ (95%CI −1.11, −0.66) and 1.26‰ (95%CI −1.50, −1.02), respectively. In model 1, for every 1,000 Yuan increase in health expenditure per capita, IMR decreased by 1.32‰ (95%CI −1.46, −1.18); for every 10 billion Yuan increase in government health expenditure, IMR decreased by 0.82‰ (95%CI −0.95, −0.70); for 1% increase in total health expenditure as a percentage of GDP, IMR decreased by 1.57‰ (95%CI −1.74, −1.39). In model 2, IMR decreased by 1.04‰ (95%CI −1.40, −0.69), 0.14‰ (95%CI −0.32, 0.05) and 0.75‰ (95%CI −0.94, −0.56), respectively.

#### Pediatric-specific health resources

In model 1, an increase of each pediatric beds per 10,000 children was associated with a decrease in IMR of 0.65‰ (95%CI −0.73, −0.57), an increase of each children’s hospital health technical personnel and maternal and child healthcare center health technical personnel was associated with a decrease in IMR of 0.46‰ (95%CI −0.67, −0.25) and 0.45‰ (95%CI −0.52, −0.38), respectively. In model 2, IMR decreased by 0.39‰ (95% CI −0.46, −0.31), 0.31‰ (95% CI −0.52, −0.10) and 0.21‰ (95% CI −0.28, −0.14), respectively. The complete model report containing the regression coefficients of each model covariate was shown in Supplementary Tables S7, S8. In the sensitivity analysis using one-year lagged independent variables, the associations between most health resource indicators and IMR remained consistent with the main findings (Supplementary Table S9).

## Discussion

This study provided evidence for optimizing resource allocation and improving infant health by analyzing the association of health resources on IMR in China. Our study found a significant negative association between health technical personnel density and IMR. This may reflect improved early detection of maternal/infant health risks and enhanced emergency response capacity in regions with higher health workforce density (11). This finding was consistent with previous studies. A global cross-country study showed that human resource intensity in health has a significant association with maternal mortality rate (MMR), IMR and under-five mortality rate (U5MR) (12). A study of 155 countries showed the number of doctors per 1,000 population was associated with a 2% decrease in IMR, and the number of nurses per 1,000 population had no significant effect on IMR (13). Studies in Iran from 2005 to 2011 showed there was a significant negative correlation between the physician density and neonatal mortality rate (NMR), U5MR, and MMR, but physician and bed densities did not have a significant effect on IMR, the reason for the difference between this result and the present study may be the need for the Iranian population to improve access to more specialized medical services and facilities (14).

For the relationship between bed density and IMR, our study showed a significant negative correlation with IMR, and this effect was greater than that for health technical personnel. This may reflect that adequately staffed beds represent both infrastructure capacity and ready-to-deploy human resources, creating a multiplier effect on emergency obstetric and infant care. A multiple regression analysis for 49 developing countries showed the association of population per hospital bed and physician with IMR was not significant (15). In a study in Brazil, hospital beds per 1,000 population was positively associated with IMR, but this effect was not significant (16), this discrepancy may reflect structural inequities in healthcare access, where historically disadvantaged racial and ethnic populations face barriers to utilizing available health resources despite adequate infrastructure. The inconsistency of findings between countries and regions may be attributed to several factors, including variations in bed utilization rates, and differences in the number of beds per health technical personnel (17, 18).

The results of this study showed that increases in health expenditure per capita and total health expenditure as a percentage of GDP were significantly associated with a reduction in IMR, while government health expenditure alone showed no significant association after adjusting for socio-economic factors. This pattern indicates that comprehensive health financing encompassing both public and private sectors may be more effective than exclusive government spending in driving IMR reduction, potentially through enabling multi-level health system improvements. Supporting this observation, another study in China found similar results, indicating that government health expenditure had a comparatively limited association with reducing IMR in comparison to other influencing factors (6). An analysis of data from 173 countries from 2004 to 2011 showed that health spending can reduce IMR levels by improving the efficiency of health services (19). A study in the Organization of the Petroleum Exporting Countries (OPEC) member countries from 2004 to 2013 showed that public health expenditure as a percentage of total health expenditure was negatively correlated with IMR (20). Analysis of data from South Asian Association for Regional Cooperation (SAARC) countries from 2000 to 2016 also showed that health expenditure had a significant association with IMR. However, analysis of SAARC countries from 1995 to 2010 showed no significant relationship between health expenditure and IMR (21, 22). The variation in the direction, intensity and targeting of health resource inputs across individual countries is largely influenced by the differing characteristics of their respective health systems, and there is a continuum of effects of health resource inputs on population health, which contributes to the complexity and inconsistency of the current results (23). The non-significant association of aggregate government health expenditure specifically may reflect that the composition and targeting of public funds, rather than total spending alone, are more critical for reducing IMR. Diagnostic tests suggested potential violations of strict exogeneity, likely reflecting reverse causality whereby past IMR levels influence subsequent resource allocation decisions—a pattern consistent with the finding that government health expenditure showed no independent association with IMR. The RESET test also indicated possible non-linearities, suggesting that threshold or diminishing-return effects may exist; future studies could explore such specifications.

The results of this study suggest that an increase in pediatric-specific health resources can significantly reduce IMR, though the magnitude of this association was smaller than that of overall health resources, which may be due to ICUs and emergency departments in general hospitals take care of some of the critically ill infants and contribute to the decrease in IMR (24–26). Research from Japan also showed the density of pediatricians was inversely related to U5MR between 2000 and 2010, and increasing human resources would be beneficial for child health (27). An analysis of China’s counties from 2015 to 2016 showed the countries with the lowest number of pediatricians per 1,000 children had a 52% higher risk of high U5MR compared to counties with the highest number of pediatricians per 1,000 children (28). A study of Guangzhou, China, showed the allocation of pediatricians and pediatric beds, as well as the efficiency of pediatric services, could reduce IMR and U5MR (29).

Our study also showed health technical personnel in children’s hospitals had a greater relationship with IMR than those in maternal and child healthcare centers. The education level and professional competency of pediatricians may affect the efficiency of their service delivery, because the proportion of pediatricians with master’s degree or above in children’s hospitals is significantly higher than that in maternal and child healthcare centers in China (30, 31). Although maternal and child healthcare centers are able to provide more convenient access and improve the availability of health services, people prefer to go to higher level general or specialized children’s hospitals, resulting in a generally low utilization of their services (32). Therefore, in order to effectively control the IMR, it is necessary to improve the capacity and quality of medical and health services in maternal and child healthcare centers, thereby increasing the overall utilization rate of children’s health resources (33, 34).

These findings highlight the importance of strategic health resource allocation for achieving the infant mortality reduction targets set by the Healthy China 2030 blueprint. The persistent provincial disparities in IMR underscore the need for differentiated investment strategies tailored to local health system capacity. Based on our findings, three priorities emerge. First, health technical personnel density should be increased in high-IMR areas to strengthen early detection and emergency response capacity. Second, bed capacity expansion should be accompanied by proportional staffing increases to maximize synergistic benefits for obstetric and infant care. Third, higher-educated pediatricians should be attracted to maternal and child healthcare centers through competitive incentives, alongside strengthened in-service training, to improve both service quality and utilization in these widely distributed but underutilized facilities.

This study has several limitations: (1) this study was based on provincial panel data and cannot control for the effects of uneven distribution of resources within the region; (2) as the missing data may not randomly distributed, observations containing missing values were deleted, resulting in a reduction in sample size and, consequently, a loss of precision and efficiency in the analyses; furthermore, the indicator for children’s hospital health technical personnel was derived from only a subset of provinces (132 province-year observations), and findings for this specific variable should be interpreted with caution rather than as nationally representative estimates; (3) this is an ecological study, and the health resource indicators represent aggregate provincial system capacity rather than infant-specific service delivery; therefore, the observed associations should be interpreted as system-level relationships, and individual-level causal inferences cannot be drawn. Additionally, crude rather than age-standardized IMR was employed because province-specific data on live births by maternal age are not available, the use of the <14 years population as the denominator for pediatric resource indicators introduces measurement noise relative to the infant-specific outcome, which likely attenuates coefficient estimates toward the null. The fixed-effects design and inclusion of socioeconomic covariates partly address this limitation by controlling for between-province demographic heterogeneity. Additionally, individual-level confounders such as maternal age, household income, and living conditions could not be directly controlled; however, the fixed-effects design mitigates bias from time-invariant provincial characteristics. Some collinearity among retained socioeconomic covariates may also persist, potentially affecting the precision of individual coefficient estimates; (4) given that the distribution of health resources across countries is shaped by the contexts of their health systems, it is essential to exercise caution when extrapolating the findings of the study to account for the inherent differences between countries in terms of policy environments, levels of health, and other pertinent factors; (5) this study focused on the association of health system factors with IMR. As a result, environmental determinants such as air pollution, forest cover, and water sanitation were not included in the analysis (35, 36). However, future studies could explore their role in IMR reduction.

Future research could further explore the heterogeneous effects of government health expenditure by disaggregating central versus local funding flows, given the non-significant association observed for aggregate government spending. Additionally, non-linear or threshold models may help clarify whether diminishing returns exist at higher levels of health resource density.

## Conclusion

This provincial panel analysis demonstrated that higher densities of health technical personnel, hospital beds, and per capita health expenditure were significantly associated with lower infant mortality rates in China from 2010 to 2020. Pediatric-specific resources showed consistent, albeit smaller, associations with IMR reduction. To accelerate progress toward the Healthy China 2030 targets, policymakers should prioritize balanced increases in both general health system capacity and pediatric-dedicated resources, with particular attention to improving the quality and utilization of maternal and child healthcare centers in underserved provinces.

## Data Availability

The raw data supporting the conclusions of this article will be made available by the authors, without undue reservation.

## Footnotes

1 Data on health workforce and hospital beds in the China Health Statistics Yearbook encompass all types of medical and health institutions, including public hospitals, private hospitals, community health centers, and township health centers, thereby reflecting the total provincial supply irrespective of ownership or funding source.
