# Supplementary material for: Panel regression analysis of the relationship between health resources and infant mortality rate in Chinese mainland provinces, 2010–2020
Source: Front Public Health. 2026 Sep 25;14:1916606. doi: 10.3389/fpubh.2026.1916606 (PMC13650015; doi:10.3389/fpubh.2026.1916606)
Supplement: Supplementary file 1 [file Supplementary_file_1.DOCX]

**Table S1.** Definitions and calculations of all variables

| Variables | Definition | Calculation method | Description of variables |
| --- | --- | --- | --- |
| Number of health technical personnel per 1,000 population | Number of health technical personnel per 1,000 population at the end of the year. | Year-end number of health technical personnel / year-end resident population × 1,000 | Health technical personnel include practicing physicians, practicing assistant physicians, registered nurses, pharmacists, inspection technicians, imaging technicians, health supervisors and trainee doctors (medicine, nursing, technical) and other health professionals. It does not include health technical personnel engaged in management work (such as president, vice president, party secretary, etc.). |
| Number of health institution beds per 1,000 population | Number of health institution beds per 1,000 population at the end of the year. | Year-end number of health institution beds / year-end resident population × 1,000 |  |
| Health expenditure per capita (1,000 Yuan) | The ratio of the total health expenditure of a certain area in a certain year to the average annual population of the area in that year. | Total health expenditure in a certain area in a certain year / (the number of permanent residents at the end of the year + the number of permanent residents at the beginning of the year) / 2) |  |
| Government health expenditure (Ten billion Yuan) | Governments at all levels for medical and health services, medical security subsidies, health and medical security administration, population and family planning affairs expenditure and other undertakings of the funds. |  |  |
| Total health expenditure as a percentage of GDP (%) | The ratio of total health expenditure to gross domestic product (GDP) in a certain region in a certain year. | Total health expenditure of a region in a certain year / GDP of the region in that year × 100 | Reflects the level of State investment in health care over a period of time and the importance attached by the Government and society as a whole to health care and the health of the population. |
| Number of pediatric beds per 10,000 children | Number of pediatric beds per 10,000 people aged < 14 years old at the end of the year. | Number of pediatric beds in medical and health institutions at the end of the year / number of permanent residents < 14 years old at the end of the year × 10,000 | Pediatrics includes pediatric surgery and child health care. Maternal and child health hospitals are included in obstetrics and gynecology, pediatrics, and children 's hospitals are included in pediatrics. |
| Number of children's hospital health technical personnel per 10,000 children | Number of children's hospital health technical personnel per 10,000 people aged < 14 years old at the end of the year. | Number of health technical personnel in children 's hospitals at the end of the year / Number of permanent residents under 14 years of age at the end of the year × 10,000 |  |
| Number of maternal and childcare service center health technical personnel per 10,000 children | Number of maternal and childcare service center health technical personnel per 10,000 people aged < 14 years old at the end of the year. | Number of health technical personnel in maternal and childcare service centers at the end of the year / Number of permanent residents under 14 years of age at the end of the year × 10,000 |  |
| Gross regional product per capita | Gross regional product calculated by the average of all permanent residents in a certain area in a certain year. | GDP of a region in a certain year / (the region at the end of the year + the number of permanent residents at the beginning of the year) / 2 ) | Regional GDP refers to the final results of the production activities of all resident units in the region in a certain period of time. Per capita regional GDP reflects the level of economic development and people 's living standards in a region. |
| Birth rate | Also known as the crude birth rate, refers to the ratio of the number of births in a certain area during the year to the average number of births in the same period. | The number of births in a certain area in a certain year / (the number of permanent residents at the end of the year + the number of permanent residents at the beginning of the year ) / 2 ) * 1,000 |  |
| Years of education per capita | The average of the total number of years of schooling received by a certain age group. | A region in a certain year (number of illiterates * 1 + number of primary school education * 6 + number of junior high school education * 9 + number of high school and technical secondary school education * 12 + number of junior college and undergraduate education * 16 ) / The total population over 6 years old in the region in that year | Reflects the education level of the new labor force in a country or region. |
| Urbanization rate | The proportion of urban resident population to the total resident population in a certain area in a certain year. | Urban resident population at year-end / resident population at year-end * 100 |  |

**Table S2** Spearman correlation analysis between all independent variables

|  | Number of health technical personnel per 1,000 population | Number of health institution beds per 1,000 population | Health expenditure per capita (Yuan) | Government health expenditure (Ten billion Yuan) | Total health expenditure as a percentage of GDP (%) | Number of pediatric beds per 10,000 children | Number of children's hospital health technical personnel per 10,000 children | Number of maternal and childcare service center health technical personnel per 10,000 children | Gross regional product per capita (Yuan) | Birth rate (‰) | Years of education per capita (year) |
| --- | --- | --- | --- | --- | --- | --- | --- | --- | --- | --- | --- |
| Number of health institution beds per 1,000 population | 0.76^*^ |  |  |  |  |  |  |  |  |  |  |
| Health expenditure per capita (Yuan) | 0.87^*^ | 0.69^*^ |  |  |  |  |  |  |  |  |  |
| Government health expenditure (Ten billion Yuan) | 0.35^*^ | 0.41^*^ | 0.32^*^ |  |  |  |  |  |  |  |  |
| Total health expenditure as a percentage of GDP (%) | 0.29^*^ | 0.49^*^ | 0.39^*^ | -0.07 |  |  |  |  |  |  |  |
| Number of pediatric beds per 10,000 children | 0.77^*^ | 0.75^*^ | 0.68^*^ | 0.27^*^ | 0.21^*^ |  |  |  |  |  |  |
| Number of children's hospital health technical personnel per 10,000 children | 0.58^*^ | 0.18^*^ | 0.60^*^ | -0.14 | 0.04 | 0.54^*^ |  |  |  |  |  |
| Number of maternal and childcare service center health technical personnel per 10,000 children | 0.64^*^ | 0.42^*^ | 0.45^*^ | 0.41^*^ | 0.02 | 0.45^*^ | 0.26^*^ |  |  |  |  |
| Gross regional product per capita (Yuan) | 0.77^*^ | 0.47^*^ | 0.80^*^ | 0.47^*^ | -0.15^*^ | 0.61^*^ | 0.64^*^ | 0.50^*^ |  |  |  |
| Birth rate (‰) | -0.38^*^ | -0.27^*^ | -0.32^*^ | -0.14^*^ | 0.17^*^ | -0.48^*^ | -0.51^*^ | -0.26^*^ | -0.40^*^ |  |  |
| Years of education per capita (year) | 0.65^*^ | 0.38^*^ | 0.58^*^ | 0.23^*^ | -.147** | 0.57^*^ | 0.76^*^ | 0.48^*^ | 0.69^*^ | -0.67^*^ |  |
| Urbanization rate (%) | 0.68^*^ | 0.34^*^ | 0.65^*^ | 0.26^*^ | -.279** | 0.60^*^ | 0.82^*^ | 0.50^*^ | 0.87^*^ | -0.62^*^ | .83^*^ |

*Significant correlation at the 0.05 level (two-tailed)

**Table S3.** Variance Inflation Factor analysis between all independent variables

| Variable | VIF | Tolerance |
| --- | --- | --- |
| Gross regional product per capita (10,000 Yuan) | 18.023 | 0.055 |
| Health expenditure per capita (1,000 Yuan) | 15.467 | 0.065 |
| Urbanization rate (%) | 10.81 | 0.093 |
| Number of health technical personnel per 1,000 population | 8.841 | 0.113 |
| Years of education per capita (year) | 6.097 | 0.164 |
| Total health expenditure as a percentage of GDP (%) | 4.841 | 0.207 |
| Number of health institution beds per 1,000 population | 4.08 | 0.245 |
| Number of pediatric beds per 10,000 children | 3.578 | 0.279 |
| Number of maternal and child healthcare center health technical personnel per 10,000 children | 2.744 | 0.364 |
| Birth rate (‰) | 2.251 | 0.444 |
| Number of children's hospital health technical personnel per 10,000 children | 2.207 | 0.453 |
| Government health expenditure (Ten billion Yuan) | 2.074 | 0.482 |

**Table S6** ADF test results for dependent and target independent variables

| Variable | Type | lag | ADF | *P* value |
| --- | --- | --- | --- | --- |
| Infant mortality rate | No drift no trend | 0 | -3.33 | 0.01 |
|  |  | 1 | -3.10 | 0.01 |
|  |  | 2 | -2.82 | 0.01 |
|  |  | 3 | -2.64 | 0.01 |
|  |  | 4 | -2.33 | 0.02 |
|  |  | 5 | -2.11 | 0.04 |
|  | With drift no trend | 0 | -6.32 | 0.01 |
|  |  | 1 | -6.15 | 0.01 |
|  |  | 2 | -5.75 | 0.01 |
|  |  | 3 | -5.65 | 0.01 |
|  |  | 4 | -5.16 | 0.01 |
|  |  | 5 | -4.80 | 0.01 |
|  | With drift and trend | 0 | -6.61 | 0.01 |
|  |  | 1 | -6.46 | 0.01 |
|  |  | 2 | -6.08 | 0.01 |
|  |  | 3 | -5.99 | 0.01 |
|  |  | 4 | -5.47 | 0.01 |
|  |  | 5 | -5.11 | 0.01 |
| Number of health technical personnel per 1,000 population | No drift no trend | 0 | -2.07 | 0.04 |
|  |  | 1 | -1.67 | 0.09 |
|  |  | 2 | -1.55 | 0.12 |
|  |  | 3 | -1.49 | 0.14 |
|  |  | 4 | -1.30 | 0.22 |
|  |  | 5 | -1.15 | 0.27 |
|  | With drift no trend | 0 | -7.89 | 0.01 |
|  |  | 1 | -6.83 | 0.01 |
|  |  | 2 | -6.65 | 0.01 |
|  |  | 3 | -6.87 | 0.01 |
|  |  | 4 | -6.25 | 0.01 |
|  |  | 5 | -5.83 | 0.01 |
|  | With drift and trend | 0 | -7.87 | 0.01 |
|  |  | 1 | -6.81 | 0.01 |
|  |  | 2 | -6.63 | 0.01 |
|  |  | 3 | -6.85 | 0.01 |
|  |  | 4 | -6.23 | 0.01 |
|  |  | 5 | -5.83 | 0.01 |
| Number of health institution beds per 1,000 population | No drift no trend | 0 | -1.75 | 0.08 |
|  |  | 1 | -1.74 | 0.08 |
|  |  | 2 | -1.58 | 0.11 |
|  |  | 3 | -1.39 | 0.18 |
|  |  | 4 | -1.21 | 0.25 |
|  |  | 5 | -1.06 | 0.30 |
|  | With drift no trend | 0 | -8.08 | 0.01 |
|  |  | 1 | -8.88 | 0.01 |
|  |  | 2 | -8.77 | 0.01 |
|  |  | 3 | -8.55 | 0.01 |
|  |  | 4 | -8.19 | 0.01 |
|  |  | 5 | -7.88 | 0.01 |
|  | With drift and trend | 0 | -8.10 | 0.01 |
|  |  | 1 | -8.93 | 0.01 |
|  |  | 2 | -8.86 | 0.01 |
|  |  | 3 | -8.66 | 0.01 |
|  |  | 4 | -8.31 | 0.01 |
|  |  | 5 | -8.02 | 0.01 |
| Health expenditure per capita | No drift no trend | 0 | -3.74 | 0.01 |
|  |  | 1 | -3.35 | 0.01 |
|  |  | 2 | -3.05 | 0.01 |
|  |  | 3 | -2.78 | 0.01 |
|  |  | 4 | -2.57 | 0.01 |
|  |  | 5 | -2.28 | 0.02 |
|  | With drift no trend | 0 | -8.18 | 0.01 |
|  |  | 1 | -7.82 | 0.01 |
|  |  | 2 | -7.61 | 0.01 |
|  |  | 3 | -7.40 | 0.01 |
|  |  | 4 | -7.31 | 0.01 |
|  |  | 5 | -6.90 | 0.01 |
|  | With drift and trend | 0 | -8.17 | 0.01 |
|  |  | 1 | -7.81 | 0.01 |
|  |  | 2 | -7.60 | 0.01 |
|  |  | 3 | -7.39 | 0.01 |
|  |  | 4 | -7.29 | 0.01 |
|  |  | 5 | -6.89 | 0.01 |
| Government health expenditure | No drift no trend | 0 | -3.94 | 0.01 |
|  |  | 1 | -3.29 | 0.01 |
|  |  | 2 | -3.03 | 0.01 |
|  |  | 3 | -2.86 | 0.01 |
|  |  | 4 | -2.74 | 0.01 |
|  |  | 5 | -2.52 | 0.01 |
|  | With drift no trend | 0 | -7.51 | 0.01 |
|  |  | 1 | -6.56 | 0.01 |
|  |  | 2 | -6.29 | 0.01 |
|  |  | 3 | -6.16 | 0.01 |
|  |  | 4 | -6.14 | 0.01 |
|  |  | 5 | -5.82 | 0.01 |
|  | With drift and trend | 0 | -7.61 | 0.01 |
|  |  | 1 | -6.66 | 0.01 |
|  |  | 2 | -6.40 | 0.01 |
|  |  | 3 | -6.28 | 0.01 |
|  |  | 4 | -6.27 | 0.01 |
|  |  | 5 | -5.95 | 0.01 |
| Total health expenditure as a percentage of GDP | No drift no trend | 0 | -1.79 | 0.07 |
|  |  | 1 | -1.62 | 0.10 |
|  |  | 2 | -1.60 | 0.11 |
|  |  | 3 | -1.45 | 0.16 |
|  |  | 4 | -1.37 | 0.19 |
|  |  | 5 | -1.22 | 0.24 |
|  | With drift no trend | 0 | -6.82 | 0.01 |
|  |  | 1 | -6.57 | 0.01 |
|  |  | 2 | -6.77 | 0.01 |
|  |  | 3 | -6.50 | 0.01 |
|  |  | 4 | -6.49 | 0.01 |
|  |  | 5 | -6.05 | 0.01 |
|  | With drift and trend | 0 | -6.83 | 0.01 |
|  |  | 1 | -6.58 | 0.01 |
|  |  | 2 | -6.79 | 0.01 |
|  |  | 3 | -6.53 | 0.01 |
|  |  | 4 | -6.53 | 0.01 |
|  |  | 5 | -6.09 | 0.01 |
| Number of pediatric beds per 10,000 children | No drift no trend | 0 | -1.61 | 0.10 |
|  |  | 1 | -1.77 | 0.08 |
|  |  | 2 | -1.45 | 0.16 |
|  |  | 3 | -1.34 | 0.20 |
|  |  | 4 | -1.18 | 0.26 |
|  |  | 5 | -1.08 | 0.29 |
|  | With drift no trend | 0 | -6.27 | 0.01 |
|  |  | 1 | -7.21 | 0.01 |
|  |  | 2 | -6.28 | 0.01 |
|  |  | 3 | -6.20 | 0.01 |
|  |  | 4 | -5.78 | 0.01 |
|  |  | 5 | -5.58 | 0.01 |
|  | With drift and trend | 0 | -6.27 | 0.01 |
|  |  | 1 | -7.21 | 0.01 |
|  |  | 2 | -6.29 | 0.01 |
|  |  | 3 | -6.21 | 0.01 |
|  |  | 4 | -5.78 | 0.01 |
|  |  | 5 | -5.58 | 0.01 |
| Number of children's hospital health technical personnel per 10,000 children | No drift no trend | 0 | -2.70 | 0.01 |
|  |  | 1 | -2.52 | 0.01 |
|  |  | 2 | -2.30 | 0.02 |
|  |  | 3 | -2.47 | 0.02 |
|  |  | 4 | -1.98 | 0.05 |
|  | With drift no trend | 0 | -3.62 | 0.01 |
|  |  | 1 | -3.46 | 0.01 |
|  |  | 2 | -3.24 | 0.02 |
|  |  | 3 | -3.54 | 0.01 |
|  |  | 4 | -2.96 | 0.04 |
|  | With drift and trend | 0 | -3.63 | 0.03 |
|  |  | 1 | -3.48 | 0.05 |
|  |  | 2 | -3.26 | 0.08 |
|  |  | 3 | -3.57 | 0.04 |
|  |  | 4 | -2.99 | 0.16 |
| Number of maternal and childcare service center health technical personnel per 10,000 children | No drift no trend | 0 | -2.29 | 0.02 |
|  |  | 1 | -2.05 | 0.04 |
|  |  | 2 | -1.91 | 0.06 |
|  |  | 3 | -1.98 | 0.05 |
|  |  | 4 | -1.72 | 0.09 |
|  |  | 5 | -1.54 | 0.13 |
|  | With drift no trend | 0 | -6.28 | 0.01 |
|  |  | 1 | -5.96 | 0.01 |
|  |  | 2 | -5.83 | 0.01 |
|  |  | 3 | -6.34 | 0.01 |
|  |  | 4 | -5.84 | 0.01 |
|  |  | 5 | -5.54 | 0.01 |
|  | With drift and trend | 0 | -6.35 | 0.01 |
|  |  | 1 | -6.04 | 0.01 |
|  |  | 2 | -5.93 | 0.01 |
|  |  | 3 | -6.47 | 0.01 |
|  |  | 4 | -5.98 | 0.01 |
|  |  | 5 | -5.70 | 0.01 |

**P* value = 0.01 means *P* value <= 0.01

**Table S5.** Diagnostic tests for panel regression model assumptions

| Test | Statistic | *P* value |
| --- | --- | --- |
| Heteroskedasticity (Breusch-Pagan) | 39.49 | 0 |
| Serial correlation (Wooldridge) | 40.825 | 0 |
| Strict exogeneity (F‑test of lead variables) | 2.247 | 0.0177 |
| Functional form (RESET) | 138.137 | 0 |

**Table S6.** Individual lead variable coefficients from strict exogeneity test

| Lead variable | Coefficient | *P* value |
| --- | --- | --- |
| Number of health technical personnel per 1,000 population | -0.11 | 0.76 |
| Number of health institution beds per 1,000 population | 0.11 | 0.86 |
| Health expenditure per capita (1,000 Yuan) | -0.65 | 0.34 |
| Government health expenditure (Ten billion Yuan) | 0.98 | 0.05 |
| Total health expenditure as a percentage of GDP (%) | -0.69 | 0.01 |
| Number of pediatric beds per 10,000 children | -0.10 | 0.43 |
| Number of children's hospital health technical personnel per 10,000 children | -0.43 | 0.61 |
| Number of maternal and child healthcare center health technical personnel per 10,000 children | 0.00 | 0.99 |
| Gross regional product per capita (10,000 Yuan) | 1.26 | 0.02 |
| Birth rate (‰) | -0.33 | 0.04 |
| Years of education per capita (year) | -1.31 | 0.17 |

**Table S7** Regression results of China's health resources on infant mortality rate from 2010 to 2020

| Variables | Model 1 | | | Model 2 | | |
| --- | --- | --- | --- | --- | --- | --- |
|  | Fixed effect (t value) | Random effect (z value) | Hausman test | Fixed effect (t value) | Random effect (z value) | Hausman test |
| Number of health technical personnel per 1,000 population | **-1.63*(-17.54)** | -1.59*(-17.41) | 0.02 | **-0.88*(-7.65)** | -0.89*(-7.66) | <0.01 |
| Number of health institution beds per 1,000 population | **-1.96*(-20.61)** | -1.93*(-20.2) | <0.01 | -1.27*(-9.9) | **-1.26*(-10.44)** | 0.42 |
| Health expenditure per capita (1,000 Yuan) | -1.34*(-18.25) | **-1.32*(-18.2)** | 0.09 | -1.07*(-5.34) | **-1.04*(-5.76)** | 0.09 |
| Government health expenditure (Ten billion Yuan) | -0.83*(-12.76) | **-0.82*(-13.00)** | 0.43 | **-0.14^†^(-1.44)** | -0.31*(-3.68) | <0.01 |
| Total health expenditure as a percentage of GDP (%) | **-1.57*(-17.44)** | -1.29*(-13.27) | <0.01 | -0.86*(-7.56) | **-0.75*(-7.77)** | 0.05 |
| Gross regional product per capita (10,000 Yuan) |  |  |  | **-0.26* (-2.39)^a^** | -0.41* (-3.93)^a^ |  |
|  |  |  |  | -0.19 (-1.81)^b^ | **-0.29* (-2.98)^b^** |  |
|  |  |  |  | 0.32 (1.66)^c^ | **0.12 (0.70)^c^** |  |
|  |  |  |  | **-0.35*(-2.47)^d^** | -0.40*(-3.13)^d^ |  |
|  |  |  |  | -0.29* (-2.73)^e^ | **-0.42* (-4.21)^e^** |  |
| Birth rate (‰) |  |  |  | **0.23* (2.89)^a^** | 0.22* (2.77)^a^ |  |
|  |  |  |  | 0.23* (2.99 )^b^ | **0.21* (2.78)^b^** |  |
|  |  |  |  | 0.25* (3.00)^c^ | **0.23* (2.85)^c^** |  |
|  |  |  |  | **0.28*(3.42)^d^** | 0.26*(3.12)^d^ |  |
|  |  |  |  | 0.20* (2.58)^e^ | **0.21* (2.73)^e^** |  |
| Years of education per capita (year) |  |  |  | **-2.62* (-4.79)^a^** | -1.39* (-3.27)^a^ |  |
|  |  |  |  | -2.15* (-4.07)^b^ | **-1.47* (-3.72)^b^** |  |
|  |  |  |  | -2.99* (-5.25)^c^ | **-1.75* (-4.24)^c^** |  |
|  |  |  |  | **-3.70*(-6.51)^d^** | -1.96*(-4.69)^d^ |  |
|  |  |  |  | -2.35* (-4.25)^e^ | **-1.79* (-4.55)^e^** |  |

a. The target independent variable was the health technical personnel.

b. The target independent variable was the health institution beds.

c. The target independent variable was the health expenditure per capita.

d. The target independent variable was the government health expenditure.

e. The target independent variable was the total health expenditure as a percentage of GDP.

*P<0.05

†P=0.15

‡The bold body represents the model selected after Hausman test.

**Table S8** Regression results of China's pediatric health resources on infant mortality rate from 2010 to 2020

| Variable | Model 3 | | | Model 4 | | |
| --- | --- | --- | --- | --- | --- | --- |
|  | Fixed effect (t value) | Random effect (z value) | Hausman test | Fixed effect (t value) | Random effect (z value) | Hausman test |
| Number of pediatric beds per 10,000 children | **-0.65* (-16.57)** | -0.63* (-16.35) | <0.01 | **-0.39* (-9.60)** | -0.38* (-9.63) | <0.01 |
| Number of children's hospital health technical personnel per 10,000 children | -0.53*(-4.14) | **-0.46* (-4.30)** | 0.31 | **-0.31* (-2.86)** | -0.20 (-1.89) | 0.04 |
| Number of maternal and childcare service center health technical personnel per 10,000 children | -0.46* (-11.90) | **-0.45* (-12.37)** | 0.47 | **-0.21* (-6.02)** | -0.23* (-6.69) | <0.01 |
| Gross regional product per capita (10,000 Yuan) |  |  |  | **-0.39* (-3.82)^a^**  **-0.30 (-1.44)^b^**  **-0.43* (-3.87)^c^** | -0.51* (-5.43)^a^  -0.60* (-2.99)^b^  -0.59* (-5.95)^c^ |  |
| Birth rate (‰) |  |  |  | **0.31* (4.09)^a^**  **0.21 (-1.28)^b^**  **0.30* (3.63)^c^** | 0.27* (3.62)^a^  -0.01 (-0.07)^b^  0.28* (3.49)^c^ |  |
| Years of education per capita (year) |  |  |  | **-2.20* (-4.14)^a^**  **-3.78* (-3.55)^b^**  **-3.01* (-5.40)^c^** | -1.26* (-2.99)^a^  -1.68 (-1.71)^b^  -1.52* (-3.53)^c^ |  |

a. The target independent variable was the pediatric beds.

b. The target independent variable was the children's hospital health technical personnel.

c. The target independent variable was the maternal and childcare service center health technical personnel.

**P* < 0.05

‡The bold body represents the model selected after Hausman test

**Table S9**. Sensitivity analysis of one-year lagged health resources on infant mortality rate (adjusted Model 2)

| Variable | Model selected | β | 95% CI | P-value | N |
| --- | --- | --- | --- | --- | --- |
| Health technical personnel per 1,000 population (lagged) | Fixed Effects | -0.89 | -1.14, -0.63 | <0.001 | 341 |
| Hospital beds per 1,000 population (lagged) | Random Effects | -1.26 | -1.55, -0.97 | <0.001 | 341 |
| Health expenditure per capita (lagged) | Random Effects | -1.04 | -1.75, -0.33 | 0.004 | 341 |
| Government health expenditure (lagged) | Fixed Effects | -0.14 | -0.54, 0.26 | 0.5 | 333 |
| Total health expenditure as % of GDP (lagged) | Random Effects | -0.75 | -0.97, -0.53 | <0.001 | 333 |
| Pediatric beds per 10,000 children (lagged) | Fixed Effects | -0.39 | -0.47, -0.30 | <0.001 | 341 |
| Children's hospital personnel per 10,000 children (lagged) | Fixed Effects | -1.17 | -1.96, -0.39 | 0.004 | 132 |
| MCH center personnel per 10,000 children (lagged) | Fixed Effects | -0.03 | -0.06, -0.00 | 0.032 | 132 |

*All models adjusted for GDP per capita, birth rate, and years of education per capita. Hausman test selected between fixed-effects and random-effects models. N = effective province-year observations.

**Figure S1** (A-H) Scatterplot of target independent variable on infant mortality rate
